# A network-based approach for predicting Hsp27 knock-out targets in mouse skeletal muscles

**DOI:** 10.5936/csbj.201303008

**Published:** 2013-08-14

**Authors:** Malek Kammoun, Brigitte Picard, Joëlle Henry-Berger, Isabelle Cassar-Malek

**Affiliations:** a INRA, UMR1213 Herbivores, F-63122 Saint-Genès-Champanelle, France; b Clermont University, VetAgro Sup, UMR1213 Herbivores, BP 10448, F-63000, Clermont-Ferrand, France; c UMR CNRS - Blaise Pascal University 6547, F-63177 Aubière Cedex, France

**Keywords:** Bioinformatics, Tenderness, Muscle, Interactome, Hsp27, HspB1-null mice

## Abstract

Thanks to genomics, we have previously identified markers of beef tenderness, and computed a bioinformatic analysis that enabled us to build an interactome in which we found Hsp27 at a crucial node. Here, we have used a network-based approach for understanding the contribution of Hsp27 to tenderness through the prediction of its interactors related to tenderness. We have revealed the direct interactors of Hsp27. The predicted partners of Hsp27 included proteins involved in different functions, e.g. members of Hsp families (Hsp20, Cryab, Hsp70a1a, and Hsp90aa1), regulators of apoptosis (Fas, Chuk, and caspase-3), translation factors (Eif4E, and Eif4G1), cytoskeletal proteins (Desmin) and antioxidants (Sod1). The abundances of 15 proteins were quantified by Western blotting in two muscles of HspB1-null mice and their controls. We observed changes in the amount of most of the Hsp27 predicted targets in mice devoid of Hsp27 mainly in the most oxidative muscle. Our study demonstrates the functional links between Hsp27 and its predicted targets. It suggests that Hsp status, apoptotic processes and protection against oxidative stress are crucial for *post-mortem* muscle metabolism, subsequent proteolysis, and therefore for beef tenderness.

## Introduction

Tenderness, flavour, juiciness, and marbling are very important attributes in the determination of beef quality even if payment on the basis of beef quality exists only in Australia at this moment. Among these attributes, there is specific attention to tenderness, which is the top priority quality attribute in beef [1]. A better control of beef tenderness is of major importance for beef producers and retailers in order to satisfy the consumers’ requirement for a consistently satisfactory product [2]. For this reason, the beef industry is looking for biological markers that would identify live animals with desirable quality attributes, in order to orientate them towards the most appropriate production systems. However, tenderness is highly variable partly due to the nature of muscle, which is a complex biological structure, consisting of fibres, adipocytes and connective tissue with different properties [3, 4]. Tenderness is also highly dependent on mechanisms occurring during the *post-mortem* transformation of muscle [5].

Transcriptomic and proteomic studies including ours [6, 7] have attempted to identify gene affecting phenotypic differences for tenderness in cattle using high-density microarrays and two-dimensional electrophoresis [6]. They have identified some potential biological markers of beef tenderness in different production systems. These biomarkers are involved in a lot of different cellular pathways such as muscle contraction, stress reactions, glycolysis and apoptosis [8]. In order to further understand the functional relationships between these markers that may participate in controlling tenderness, we computed a bioinformatic analysis [9]. It allowed the construction of a first “tenderness network” consisting of 330 proteins based on 24 initial biomarkers of beef tenderness. In this network, heat shock proteins and especially the Hsp27 were found at crucial nodes [9]. Hsp27 is encoded by the HspB1 gene and belongs to the small heat shock family also called Hsp20 family, comprising the Hsp20, Hsp27, and αβ-crystallin. Interestingly, several studies have shown that Hsp27 expression is correlated with tenderness and could be used as a tenderness biomarker [6, 10–12]. Its role in tenderness could be achieved partly through apoptosis and be correlated with its phosphorylation and oligomeric size [13].

Hence, the aim of the present study was to analyze the consequences of the targeted invalidation of the HspB1 gene on the proteins interacting with Hsp27 and linked to beef tenderness. We performed a network analysis to reveal the partner proteins of Hsp27. Then, we analyzed their abundance in the muscle of HspB1-null mice and their controls. The study enabled the identification of several pathways potentially involved in the determination of tenderness.

## Materials and methods

### Bio-informatics

The first part of the work was devoted to the identification of proteins that interact with Hsp27 according to information stored and shared in bioinformatic databases. This was performed using the software for systems biology Pathway Studio (Ariadne Genomics). Pathway Studio helps to interpret experimental data in the context of pathways, gene regulation networks, protein interaction maps, and to automatically update pathways with newly published facts using MedScan technology (www.elsevier.com). The Medscan reader extracts the relationship information from literature. We used the ResNet Mammalian (human, rat and mouse) database which contained the latest information extracted from the literature and from published high-throughput experiments. The approach was to build a network centred on Hsp27 interactors also called nearest neighbours. The filter options used were “protein” as entity type and “regulation” and “direct regulation” as applicable relation types. Then, the intersection between the Hsp27 neighbours and the list of 330 proteins from a previous tenderness network [9] was computed to get a list of Hsp27 interactors putatively linked to tenderness.

### Animals and experimental procedure

In this study we used a constitutive knock out by gene deletion of HspB1 in mice (HspB1-null mice. This was achieved through targeted insertion (homologous recombination) as described in Kammoun *et al*. [14]. About 100% of the HspB1 coding sequence gene was replaced by bacterial vector obtained from BMQ BAC library (Mouse Micer vector set 369N20). The commercial heterozygous ES cells (HspB1 ^-/+^) were microinjected into the blastocoels of mouse embryos. Embryos that received ES cells were then implanted into surrogate mothers. The resulting chimeras with a high percentage of agouti coat color were mated to wild type C57BL/6 mice to generate F1 offspring. All experiments using homozygous (HspB1 ^+/+^), heterozygous (HspB1 ^-/+^), or HspB1 homozygous null mice (HspB1 ^-/-^) were performed on C57BL/6 background. The F2 offspring were mated in order to amplify the three strains. Mice were housed at the experimental plant of nutrition and microbiology of the National Institute of Agronomic Research (INRA-France), in a temperature and humidity controlled room under a 12-hour light and dark cycle. They were fed *ad libitum*. Ten males were selected to constitute 2 experimental groups. Experimental procedures and animal holding respected French animal protection legislation, including licensing of experimenters. They were controlled and approved by the French Veterinary Services (agreement number CE 84-12).

### Muscle samples

The HspB1-null mice were sacrificed at 12 weeks postnatal. Two muscles with different composition in fibre types were collected, namely the m. *Soleus* (slow oxidative) and the m. *Tibialis Anterior* (fast glycolytic) [15]. Muscle samples were taken immediately after sacrifice, frozen in liquid nitrogen and kept at -80 °C until protein extraction. Total protein extractions were performed according to Bouley et al. [16] in a denaturation/extraction buffer (8.3 M urea, 2 M thiourea, 1% DTT, 2% CHAPS) and stored at -20°C until use. The protein concentration was determined by spectrophotometry with the Bradford assay [17].

### Immunological protein quantification

The conditions for use and specificity of primary antibodies against candidate proteins were assessed by Western blotting in order to check the specificity of all the antibodies. An antibody was considered specific when its target bands were detected at the expected molecular weight. Fourteen primary antibodies were tested for their specificity and their optimal dilution ratios were determined. Conditions used and suppliers for all primary antibodies are reported in Table 1. Secondary fluorescent-conjugated IRDye 800CW antibodies were supplied by LI-COR Biosciences (Lincoln, NE, USA) and used at 1/20000.

**Table 1:** Suppliers and conditions for each antibody used in this study.

Target protein	Protein name	Primary antibody type	References	Dilution
Hsp27	Heat schock protein 27	Monoclonal	Santa Cruz: SC13132	1/1000
Hsp20	Heat shock protein 20	Monoclonal	Santa Cruz: SC51955	1/200
Cryab	Crystallin, alpha B	Monoclonal	Enzo: SPA-222	1/2000
Hspbap1	Heat shock protein 27-associated protein 1	Polyclonal	Santa Cruz: SC-99444	1/4000
Hsp40	Heat shock protein 40	Monoclonal	Santa Cruz: SC-56400	1/400
Hsp70a1a	Heat shock protein 70 1A	Monoclonal	R&D Systems: #242707	1/500
Hsp90aa1	Heat shock protein 90-alpha	Monoclonal	R&D Systems: #341320	1/500
Fas	Tumour necrosis factor receptor superfamily member 6, TRAF6	Polyclonal	R&D Systems: #AF 435	1/500
Chuk	Inhibitor of nuclear factor Kappa-B kinase subunit alpha	Polyclonal	Tebu-bio: E11-0441A	1/1000
Sod1	Superoxide dismutase	Polyclonal	ACRIS: APO3021PU-N	1/2000
Casp3	Caspase-3	Polyclonal	Santa Cruz: SC-7148	1/500
Cycs	Cytochrome c	Polyclonal	Tebu-bio: PAB 8027	1/10000
Eif4E	Eukaryotic translation initiation factor 4E	Monoclonal	R&D Systems: clone 299910	1/250
Eif4G1	Eukaryotic translation initiation factor 4 gamma 1	Monoclonal	Tebu-bio: H00001981-M10	1/1000
Des	Desmin	Monoclonal	DAKO: D33 M0760	1/250

The abundance of candidate proteins was measured by Western blotting in the m. *Soleus* and the m. *Tibialis Anterior* of HspB1-null mice *vs* their control littermates. Fifteen µg of proteins were separated by gel electrophoresis using SDS-PAGE for 2 hr according to the Laemmli method [18]. After migration, the proteins were transferred onto PVDF transfer membrane Millipore (Bedford, MA01730, USA). Membranes were then blocked with 5% non-fat milk in TBS1 x buffer containing (blocking solution) and incubated under gentle agitation all night at room temperature in the presence of the primary antibodies. Then the membranes were incubated at 37°C for 30 minutes with the secondary fluorochrome-conjugated LICOR-antibody. Infrared fluorescence detection was then used for protein quantification. Membranes were scanned by the scanner Odyssey (LI-COR Biosciences) at 800 nm. Band volumes were quantified in the images using ImageQuant TL v 7.0.1.0 software (Amersham). Protein abundance for each sample is given in arbitrary units.

### Statistical analysis

The differences in muscle protein abundance between HspB1-null mice (n=5) and their controls (n=5) were assessed by analysis of variance (ANOVA) using XLSTAT Software [19]. The effects tested in the model included muscle (M), genotype (G), and muscle*X*genotype interaction (M*X*G). Results are expressed as the LS-mean ± standard error of mean (SEM). A difference between groups was considered significant when P<0.05.

## Results

### Network analysis

The first step of our study was to build a network of the Hsp27 nearest neighbours (direct interactors) using the Pathway Studio software according to the information stored and shared in bioinformatics databases of mammalian experiments. As shown in Table 2, the network comprised 34 proteins predicted as direct interactors of Hsp27, but was not a hub in the tenderness network [9]. A gene ontology analysis indicated that these proteins belonged to different biological processes such as the response to heat, apoptotic process, and response to oxidative stress.

**Table 2:** Protein names, gene names and references in *Mus musculus* of 34 nearest neighbours of Hsp27.

Protein	Protein name	Protein ID *SWISSPROT*	Gene	Gene ID *NCBI*	Gene Ontology	References
Hspb6	Heat shock protein 20	Q5EBG6	hspb6	243912	Regulation of muscle contraction	[20]
Hspb8	Heat shock protein 22	Q9JK92	hspb8	80888	Response to stress	[21]
Hspb1	Heat shock protein 27	P14602	hspb1	15507	Regulation of apoptotic process	[22]
Hspbap1	Heat shock protein 27-associated protein 1	Q8BK58	hspbap1	66667	Response to stress	[23]
Hsp90aa1	Heat shock protein 90-alpha	A0PJ91	hsp90aa1	15519	Response to stress	[24]
Ins2	Insulin-2	P01326	ins2	16334	Regulation of apoptotic process	[25]
Vcl	Vinculin	Q64727	vcl	22330	Regulation of cell migration and adhesion	[26, 30]
Des	Desmin	P31001	des	13346	Muscle development	[27]
Casp3	Caspase-3	P70677	casp3	12367	Regulation of apoptotic process	[28]
Cald1	Caldesmon1	Q8VCQ8	cald1	109624	Regulation of muscle contraction	[29]
Cycs	Cytochrome c	P62897	cycs	13063	Regulation of apoptotic process	[30]
Lalba	Alpha-lactalbumin	P29752	lalba	16770	Lactose biosynthetic process	[31]
Akt1	Protein kinase B alpha	P31750	akt1	11651	Regulation of apoptotic process	[32]
Sod1	Superoxide dismutase	P08228	sod1	20655	Muscle cell homeostasis	[33]
App	Amyloid beta A4 protein	P12023	app	11820	Regulation of mitotic cell cycle	[34]
fgf-2	Fibroblast growth factor 2	P15655	fgf-2	14173	Regulation of apoptotic process	[35]
Cdh1	Cadherin-1	P09803	cdh1	12550	Regulation of cell adhesion	[36]
Tnni3	Troponin I, cardiac muscle	P48787	tnni3	21954	Regulation of muscle contraction	[37]
Tnnt2	Troponin T, cardiac muscle	Q6P3Z7	tnnt2	21956	Regulation of muscle contraction	[38]
Bcl-2	Apoptosis regulator BCL-2	P10417	bcl-2	12043	Regulation of apoptotic process	[39]
Rhoa	Transforming protein RhoA	Q9QUI0	rhoa	11848	Muscle development	[40]
Traf6	TNF receptor-associated factor 6	P70196	traf6	22034	Regulation of apoptotic process	[41]
Diablo	Diablo homolog, mitochondrial	D3Z2Q3	diablo	66593	Regulation of apoptotic process	[42]
Nefl	Neurofilament light polypeptide	P08551	nefl	18039	Organization of the neurofilament	[43]
Daxx	Death domain-associated protein 6	O35613	daxx	13163	Regulation of transcription	[44]
Mapt	Microtubule-associated protein tau	P10637	mapt	17762	Regulation of microtubule polymerization	[45]
Dusp1	Dual specificity protein phosphatase 1	P28563	dusp1	19252	Regulation of apoptotic process	[46]
Msr1	Macrophage scavenger receptor types I	P30204	msr1	20288	Regulation of cholesterol storage	[47]
Apaf1	Apoptotic protease-activating factor 1	O88879	apaf1	11783	Regulation of apoptotic process	[48]
G6pdx	Glucose-6-phosphate 1-dehydrogenase	Q00612	g6pdx	14381	Response to oxidative stress	[49]
Eif4e	Eukaryotic translation initiation factor 4E	P63073	eif4e	13684	Regulation of translation	[50]
Eif4g1	Eukaryotic translation initiation factor 4 gamma 1	Q6NZJ6	eif4g1	208643	Regulation of translation	[51]
Fas	Tumour necrosis factor receptor superfamily member 6, TRAF6	P25446	fas	14102	Regulation of apoptotic process	[52]
Chuk	Inhibitor of nuclear factor kappa-B kinase subunit alpha	A0AUV3	chuk	12675	I-kappaB phosphorylation	[53]

**Figure 1:**
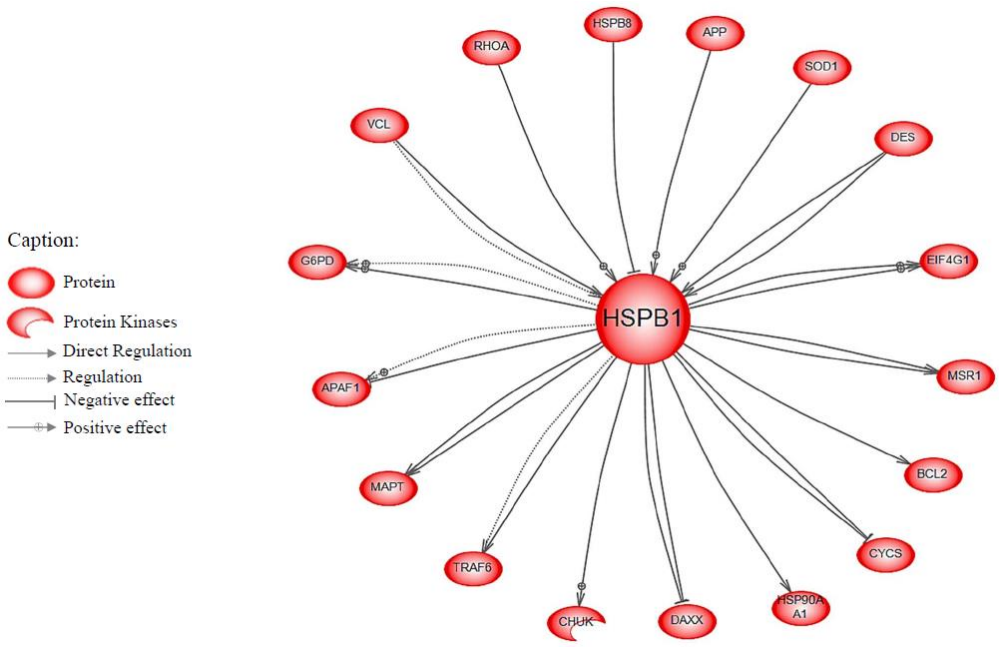
**Network of the intersection between Hsp27 neighbours (HspB1 gene) and the 330 proteins of the tenderness interactome** [11]. The protein names are presented in Table 2. The network was built using Pathway Studio. The filter options are: *protein* as an applicable entity type, *regulation* and *direct regulation* as applicable relation types.

As the initial Hsp27 network was built independently of tenderness, we performed an intersection between both networks to keep the Hsp27 interactors potentially linked to beef tenderness. Thus we compared the list of the Hsp27 neighbours with the 330 proteins of the tenderness network. The proteins in common (intersection) were then subjected to Pathway Studio analysis. This led to a second network of 17 proteins directly interacting with Hsp27 (Figure 1). The Heat shock protein 22 (Hspb8) and Heat shock protein 90 (Hsp90aa1) were the only heat shock proteins found in this network. Five proteins involved in apoptosis were also identified (Cytochrome c, Apoptosis regulator Bcl-2, TNF receptor-associated factor 6, Death domain-associated protein 6, and Apoptotic protease-activating factor 1). Some proteins (e.g. Vinculin, Desmin, Amyloid beta A4 protein, Transforming protein A, and Microtubule-associated protein) were related to muscle contraction and structure. Two other groups of proteins included anti-oxidants (Superoxide dismutase and Glucose-6-phosphate 1-dehydrogenase) and proteins involved in cellular metabolism (Macrophage scavenger receptor types I, Eukaryotic translation initiation factor gamma 1, and the Inhibitor of nuclear factor kappa-B kinase subunit alpha.

In conclusion, the network approach predicted that 17 of the 34 interactors of Hsp27 may be related to meat tenderness (Figure 1). These proteins belonged to different biological families (Heat shock proteins, apoptosis, cell protein metabolism, structure, and response to oxidative stress).

### Validation of a set of the Hsp27 predicted targets

Depending on the availability of antibodies, the abundances of 15 proteins including 12 out of these 17 interactors, the Hsp40 /Dnaja1 (a patented marker of beef toughness [54]), the Hsp70 (a well-known Hsp27 co-chaperone [55]), and Hsp27 were compared between the HspB1-null mice and control ones. As expected, the Hsp27 protein was not detectable in the muscles of the HspB1-null mice (Table 3). The statistical analysis showed a significant effect of muscle for all proteins except Hsp40, Cycs, and Eif4E, of genotype for all proteins except Hsp40, Chuk, Hspbap1 and Caspase3 (Table 3). A muscle x genotype interaction was detected for Cryab (P<0.1), Hspbap1 (P<0.1), Hsp70a1a (P<0.05), Sod1 (P<0.1), Casp3 (P<0.001), Eif4G1 (P<0.05), and Desmin (P<0.1) (Table 3).

**Table 3:** Abundance of Hsp27 interactors in the m. *Tibialis Anterior and m. Soleus* of mice.

Protein	*m. Tibialis Anterior*		*m. Soleus*		*SEM*	*Significanceof effect*

	*HspB1-null mice*	*Control mice*	*HspB1-null mice*	*Control mice*		
Hsp27	0	51879^b^	0	114175^a^	3525	M***, G***, M*x*G***
Hsp20	156240^ab^	185010^a^	98369^b^	178891^a^	12984	M^t^, G*
Cryab	450204^c^	329197^c^	6731013^a^	4918470^b^	344702	M***, G^t^, M*x*G^t^
Hspbap1	118207^a^	128968^a^	60568^b^	45241^b^	4864	M***, M*x*G^t^
Hsp40	24857^a^	26989^a^	24529^a^	16046^a^	2727	-
Hsp70a1a	16977^c^	8980^c^	357462^a^	268393^b^	7658	M***, G*, M*x*G*
Hsp90aa1	23363^b^	13718^b^	46043^a^	22752^b^	3020	M*, G**
Fas	103957^a^	88394^a^	48383^b^	27529^c^	4551	M***, G*
Chuk	57503^a^	55918^a^	13081^b^	17995^b^	1859	M***
Sod1	797670^a^	782077^a^	659073^b^	532988^c^	25989	M***, G*, M*x*G^t^
17 kDa Casp3	36436^b^	46469^a^	35280^b^	22977^c^	1724	M***, M*x*G***
Cycs	1859951^ab^	1930851^ab^	1841464^b^	2366463^a^	112016	G^t^
Eif4E	26760^ab^	23925^ab^	30538^a^	22413^b^	1460	G*
Eif4G1	141750^c^	128482^c^	429500^a^	333187^b^	9464	M***, G**, M*x*G*
Desmin	106167^b^	128907^b^	150110^b^	242959^a^	15125	M**, G*, M*x*G^t^

The abundances of 15 Hsp27 interactors were measured by Western blotting.

The protein names are presented in Table 1. Protein abundance for each sample is given in arbitrary units.

a, b, c, d LS-means with different superscripts within a row are significantly different (P<0.05).

For Caspase-3, the 17 kDa fragment was quantified.

M: muscle effect; G: genotype effect; MxG: muscle and genotype interaction

* P<0.05;

** P<0.01;

*** P<0.0001;

t tendency, P<0. 1 HspB1-null mice (n=5); control mice (n=5)

In the m. *Tibialis Anterior*, a lower abundance of the 17 kDa caspase-3 was detected in the HspB1-null mice (Table 3). A trend was observed for lower abundance of Hsp20 in the HspB1-null mice.

In the m. *Soleus* muscle, more differences were observed between HspB1-null mice and their controls than in the m. *Tibialis Anterior* (Table 3). The abundances of the Cryab, Hsp70a1a and Hsp90aa1 were higher and that of Hsp20 was lower in the HspB1-null mice. Abundances of the 17 kDa Caspase-3, and Fas were higher and Cycs was lower in the HspB1-null mice than in controls. The abundance of the translation factors Eif4E and Eif4G1 was higher in HspB1-null mice than in controls (P<0.05 and P<0.01, respectively). Lastly, Sod1 was higher (P<0.05) and Desmin was lower (P<0.01) in the HspB1-null mice.

In conclusion, we observed changes in the amount of most of the Hsp27 predicted targets in the HspB1-null mice. These changes were more marked in the oxidative muscle.

## Discussion

Our previous studies have brought out Hsp27 as a beef quality biomarker [10, 54, 56–58]. However, the relationships between the expression of HspB1 (encoding Hsp27) and tenderness are not fully understood. A positive correlation of Hsp27 protein level and shear force value in Korean cattle was shown. Recent studies with French breeds confirm that correlation of Hsp27 level may be positive or negative depending on the cattle breed [10, 59]. In order to understand Hsp27 function in muscle and its putative role in tenderness, we have used HspB1-null mice (devoid of Hsp27) as a model. Our strategy was to analyze the consequences of HspB1 targeted invalidation on the abundance of other muscle proteins related to beef tenderness. These proteins were investigated by a network-based approach that allowed a prediction of the effect of HspB1 knock-out. The prediction was borne out by a biochemical approach. Interestingly, 10 of 14 proteins were upregulated in the HspB1-null mice. The Hsp27 targets putatively related to tenderness belonged to five main protein families (Hsps, pro/anti-apoptotic factors, translation factors, cytoskeletal proteins, and antioxidants).

### Hsp status

Firstly, the approach enabled the identification of six Hsps belonging to different groups, namely the small Hsp (Cryab, Hsp20, and Hspbap1), Hsp70, and Hsp90. The Hsp status was modified, except for Hspbab1 and Hsp40, in response to Hsp27 invalidation in the m. *Soleus*. This was not observed in the m. *Tibialis Anterior*. Hsps are ubiquitously expressed molecular chaperones that are involved in the post translational folding of proteins. They promote the maturation, structural maintenance and proper regulation of specific target proteins involved for instance in cell cycle control and signal transduction. They interact dynamically with various co-chaperones that modulate their substrate recognition, ATPase cycle and chaperone function. They also play an important role in the anti-apoptotic pathway, in the inhibition of reactive oxygen species (ROS) formation and their chaperone activity ensures a good functioning of the muscle under constitutive oxidative stress conditions [60]. Cells usually overexpress Hsps in response to a multitude of insults (e.g. heat, oxidative stress, heavy metals, or cytotoxic agents among others) to prevent cell death and enable cells to survive under otherwise stressful and lethal conditions [61].

The abundance of Hsps is regulated by heat shock factors (Hsfs), the upstream transcriptional regulators of Hsps [62]. Among the Hsf family, Hsf1 is crucial for the heat shock response in mammalian organisms [63]. Under normal conditions, Hsf1 exists in a transcriptionally repressed state, associated to Hsp90 and Hsp70. The dissociation of Hsp90 and Hsp70 from Hsf1 under stress conditions leads to the activation of Hsf1. Then the monomeric Hsf1 trimerizes, phosphorylates and translocates to the nucleus where it transactivates the Hsp genes (e.g. Hsp27, Hsp70 and Hsp90) [61]. The existence of a negative feedback mechanism to return Hsf1 to its inactive monomeric state has been proposed [64]. Hsp27 exerts a feedback inhibition of Hsf1 transactivation [65]. Therefore, in the absence of Hsp27, Hsf1 would remain activated and the transcription of Hsp70 and Hsp90 genes would remain turned on. Accordingly, we showed higher abundance of Hsp70 (Hsp70a1a) and Hsp90 (Hsp90aa1) in the m. *Soleus* of HspB1-null mice. The abundance of the related small heat shock protein Cryab increased. However, Hsp20 was down-regulated in the HspB1-null mice. Compared to the other Hsps, the expression of Hsp20 probably does not depend on the action of heat shock factor (Hsf1) [66].

Altogether, these data suggest that the HspB1-null mice could adapt to the loss of Hsp27 through compensatory changes in the muscle expression of cognate members of the Hsp family. Thus Hsp27 could also play a crucial role in orchestrating Hsp abundance under physiological and unstressed conditions. However, our data were not in accordance with Huang et al. [67], who did not observe any significant differences in the basal level of several Hsps (e.g. Hsp70, Hsp90, Hsp40, and Cryab) in the muscles after HspB1 invalidation.

### Regulation of apoptosis

In our study, some proteins involved in the regulation of apoptosis were also predicted as Hsp27 targets based on our network analysis. This was validated by Western blot analysis. We detected up-regulation of pro-apoptotic proteins (e.g. active caspase-3, and Fas) in the m. *Soleus* of HspB1-null mice. These data are in agreement with the well-known anti-apoptotic effects of Hsp27 [68] and more generally of members of the small Hsp family. Hsp27 protects the cells from apoptosis by concerning with Daxx, tBid, Cytochrome c, Ikk, Caspase-3 and etc. [66, 69]. Some studies showed that overexpression of Hsp27 and Hsp20 prevents the cytochrome c activation of Caspase 9 and 3 playing a central role in the execution of apoptosis [70]. Reports have already mentioned decreased levels of procaspase-3 [71–73] in cells devoid of Hsp27. An interaction has been described between the pro-domain of procaspase-3 and Hsp27, which modulates procaspase-3 cleavage and activation [69]. Gibert et al, [74] proposed that Hsp27 could modulate procaspase-3 half-life. In the absence of Hsp27, procaspase-3 would be rapidly degraded through the ubiquitin/proteasome pathway. Accordingly, procaspase-3 tended to decrease in the m. *Tibialis Anterior* of the HspB1-null mice and was undetectable in their m. *Soleus* (data not shown).

Thus, our data suggest that the decrease in small Hsps (Hsp27 and Hsp20) with anti-apoptotic activity would increase apoptosis in the muscles of HspB1-null mice. Indeed, Hsp27 can interfere with the signals leading to apoptosis [66], at different stages of the apoptotic process (receptors, effectors, and inhibitors). Interestingly, the abundance of the inhibitor of nuclear factor kappa-B kinase subunit alpha (Ikk-α, also known as Chuk) was decreased in the HspB1-null mice. Ikk-α is part of the IκB protein kinase complex. It is the predominant form of Ikk in the mammalian cells [75] that plays an important role in regulating the NF-κB transcription factor activity. NF-κB is present in the cytoplasm in an inactive form complexed with IκB that prevents its translocation to the nucleus where it binds to DNA and induces the transcription of a number of anti-apoptotic genes [76]. Activation of NF-κB transcriptional activity has been proposed as another pathway providing for the anti-apoptotic effect of Hsp27 [66]. The phosphorylation of IκB by protein kinase promotes its ubiquitylation and proteasomal degradation. This process is enhanced by Hsp27, which forms tight complexes with ubiquitylated IκB and 26S proteasome and promotes its proteosomal degradation [66]. In our study, there were no elements to account for the reduction in Ikk-α in the absence of Hsp27.

### Translation factors

Eif4E and Eif4G, two eukaryotic translation initiation factors were identified by the network approach. Their abundances were found to be increased in the m. *Soleus* of the HspB1-null mice. This was in favour of an increase in the availability of Eif4E (the principal activator of cap-dependent translation) and Eif4G for protein translation. There are some data linking small Hsps to translation. Hsp27 specifically bounds Eif4G during heat shock, preventing assembly of the cap-initiation/Eif4F complex and trapping Eif4G in insoluble granules [77] and/or promoting a more rapid recovery of translation initiation after stress [78]. Moreover, some studies have also shown that the overexpression of Eif4E rescues cells from apoptosis [79] by inhibiting the release of cytochrome c from the mitochondria. Bcl-XL has been found to be the mediator of Eif4E-dependent anti-apoptotic signaling upstream of mitochondria. In our study, the increased Eif4E (and Eif4G) could be part of a mechanism by which transcripts are translationally activated to mitigate the stimulation of the apoptotic pathway. Thereby, the cells could survive in the absence of Hsp27.

### Regulation of the cytoskeleton

Small Hsps have been shown to be associated with the three major cytoskeletal components: microtubules, intermediate filaments and micro-filaments [80]. In our study, there was a significant decrease in the abundance of Desmin in the m. *soleus* of the HspB1-null mice. It was reported that Hsp27 protects Desmin from Calpain proteolysis [81]. Hsp20 also plays an important role in the protection of structural proteins like Desmin (intermyofibrillar cytoskeleton), Actin and Titin [9], and inhibits the formation of aggregates [82]. On the other hand, Panagopoulou et al. [83] demonstrate that Caspase mediated Desmin degradation and could act in parallel with Calpains which are known to be activated by TNF-α [84]. In the HspB1-null mice there was an increase in the abundance of TNF- α receptor associated factor (Fas) and caspase-3, which could lead to a decrease in Desmin abundance. This could have a consequence for the kinetics of *post-mortem* degradation of the ultra-structure of muscle detected in the HspB1-null mice (Kammoun *et al*., submitted).

### Protection against oxidative stress

Small heat shock proteins modulate the ability of the cells to respond to oxidative stress. For Hsp27 this effect includes a role in regulating enzymes such as the glucose-6-phosphate [80]. HspB1-null mice showed a significant increase in the abundance of the superoxide dismutase Sod1 in the m. *Soleus* compared to control mice. Sod1 is an enzyme that dismutes the superoxide anion and is involved in antioxidant defences [85]. Oxidative stress is accompanied by increased levels of toxic ROS, such as peroxides and free radicals. Overexpression of Hsp27 led to a significant decrease in basal levels of ROS and ROS production under conditions of oxidative stress [66]. In our study, the loss of Hsp27 could have led to increased basal ROS levels and subsequently to increased Sod1 levels protecting cells from antioxidant stress.

In conclusion, our study demonstrates the functional links between Hsp27 and its predicted targets as illustrated in mice devoid of Hsp27 under basal conditions (thermo neutrality, no physical or emotional stress). Particularly, changes in the abundance of these targets in HspB1-null muscles may be a mechanism to compensate for the absence of Hsp27. Our data also suggested that the apoptotic pathway may be stimulated in the HspB1-null mice through receptors, effectors, and inhibitors of apoptosis. This phenomenon being mediated by mitochondria, it may not be surprising to see the more dramatic effects in high mitochondrial content slow muscle. Additionally, Hsp27 seemed to modulate many elements of the cytoskeleton and would thus play an important role in the regulation of its dynamics and remodelling. All these elements are crucial for the tenderizing process. Based on these data, we can hypothesize that the *post-mortem* ageing and tenderizing process in beef could rely not only on proteolysis but also on regulation of apoptotic processes, and protection against oxidative stress. In the future, integration of the knowledge gained from this study could finally result in optimizing meat production through detection of desirable animals. Moreover, the effect of Hsp27 loss was detected in the slow oxidative muscle (*Soleus*) rather than in the fast glycolytic muscle (*Tibialis Anterior*). This indicated that the invalidation of HspB1 has muscle-specific effects probably in relation to the higher abundance of Hsps in the slow oxidative muscles. This is consistent with the weight assigned to Hsps in beef tenderness prediction in oxidative muscles [86].
